# Supplementary material for: Detecting 3D Vegetation Structure with the Galileo Space Probe: Can a Distant Probe Detect Vegetation Structure on Earth?
Source: PLoS One. 2016 Dec 14;11(12):e0167188. doi: 10.1371/journal.pone.0167188 (PMC5156343; doi:10.1371/journal.pone.0167188)
Supplement: S1 File — Fig A, Test comparing the Amazon and the Sahara Desert. Fig B, Landsat image showing small clouds. (PDF) [file pone.0167188.s001.pdf]

PLOS ONE

Supporting information

S Methods

To further verify our results, we use data from the MODIS Terra satellite comparing the anisotropy of a region with abundant vegetation with three-dimensional structure (the Amazon) to one without (the Sahara Desert). We use the MOD09A1 product that supplies the surface spectral reflectance as it would be measured at ground level in the absence of atmospheric scattering or absorption. This product uses the most cloud free, highest quality observations during an 8-day period for each pixel. Each pixel has associated azimuth, view, and zenith angles from whichever period used within the eight-day period. We selected regions for the Amazon who had azimuth angles close to the principle plane (near 0 azimuth) and view and zenith angles overlapping. There is much variation in solar zenith and view angles within the data because different data within the eight-day period are chosen. We calculate reflectance for 2012 (near day 185) for the Amazon (averaging all pixels in h10v09) and July 2008 - 2014 (near day 249) for the Sahara (averaging all pixels in h18v06). To eliminate the large spectral differences between the two scenes and focus just of the geometric differences, we subtract the mean reflectance at 5˚ to normalize both scenes to the same nadir brightness.

S Results

Using the MODIS satellite data, we find that the mean spectral reflectance in the NIR at 5˚ view zenith angle was ~0.20 in the Amazon and ~0.60 in the Sahara (SI Figure 1). Spectrally, the two regions are distinct. However, if the Sahara were covered with single cellular life with the same spectral properties as the Amazon, the two regions would have similar spectral properties. To simulate this, we remove the mean spectral value from both regions (SI Figure 1). Now, we can compare the change in reflectance due only to anisotropic effects. As the satellite's view angle approaches the solar angle there is a large increase in reflectance of 0.10 in the region with abundant vegetation with three-dimensional structure (the Amazon) as the shadows are obscured, but a much smaller increase in reflectance of 0.04 in the region without such life (in the Sahara). Therefore, with clear, high resolution satellite data vegetation with three-dimensional structure is clearly distinguishable from areas without three-dimensional structure but with a similar spectral signature using anisotropic effects.

S Discussion

We tested our hypothesis under ideal conditions on cloud free, high resolution data (MODIS data which removed cloudy periods) for a region largely without three-dimensional vegetation structure (the Sahara) and one with (the Amazon) (SI Figure 1). We subtracted the overall brightness for both regions, to isolate differences due only to anisotropy, which we hypothesize are an additional source of information to the spectroscopy. We found that with increasing solar zenith angle, brightness increases substantially more (by ~0.06) in our region with three-dimensional vegetation structure than our region without. In practice, this is not an ideal experiment because if the Amazon basin were stripped of its vegetation, the surface would likely be much smoother than the Sahara with fewer shadows because of the abundant rainfall and highly weathered clay surfaces in the Amazon. Therefore, the comparison between the Amazon and the Sahara likely underestimates the differences that would really exist between an area with three-dimensional vegetation structure and a region without but with similar climate. However, despite these differences, this data demonstrates there is a clear difference in reflectance between these two regions due to anisotropy, which supports our hypothesis that under the correct conditions, anisotropy can detect three-dimensional vegetation structure.

S Figures

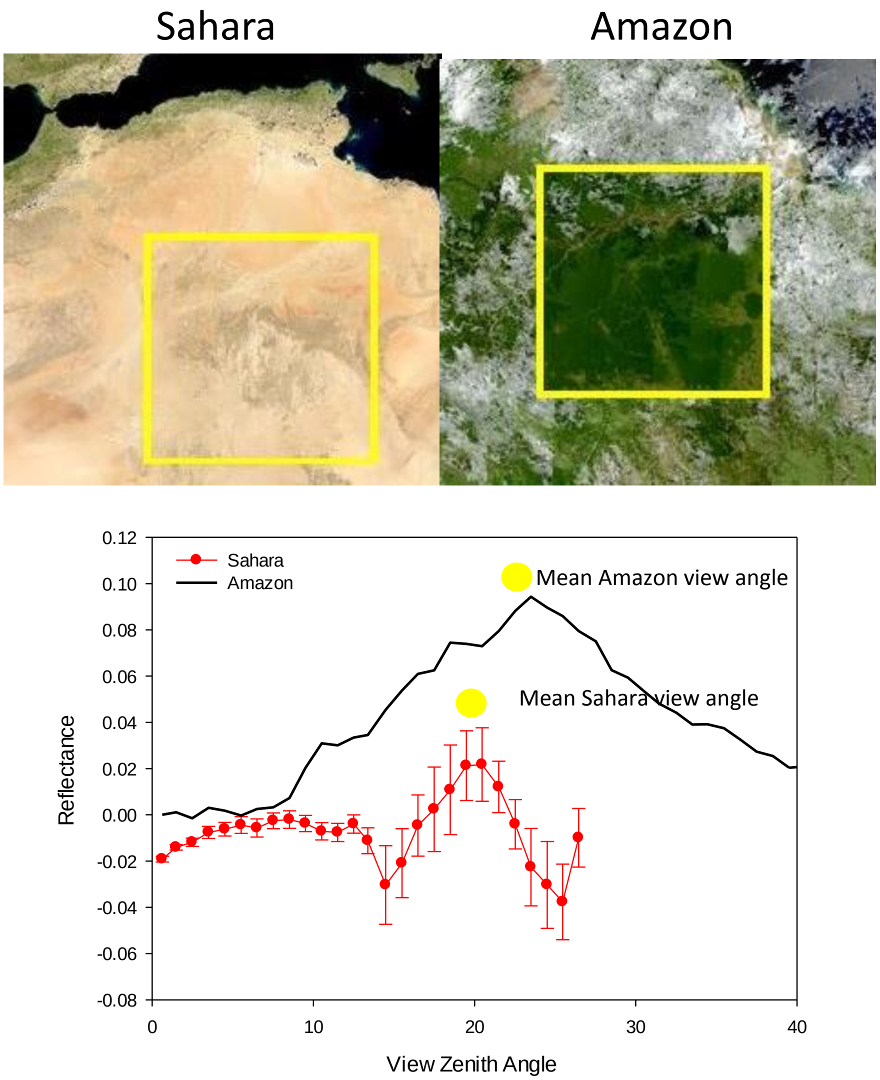

Fig A – Mean NIR reflectance for the Sahara Desert (region on top left) and the Amazon forest (region on top right) subtracting the mean spectral value at 5˚ view zenith angle (0.20 for the Amazon and 0.60 for the Sahara). Yellow dots show the mean solar zenith angle for the two regions. Error bars are standard errors between several years for the Sahara Desert. There are no error bars for the Amazon because there are fewer cloud free images. Both images are close to the principle plane with a mean azimuth angle of 10.2 ±7 (sd) for the Amazon and 7.5 ±10 (sd) for the Sahara Desert.

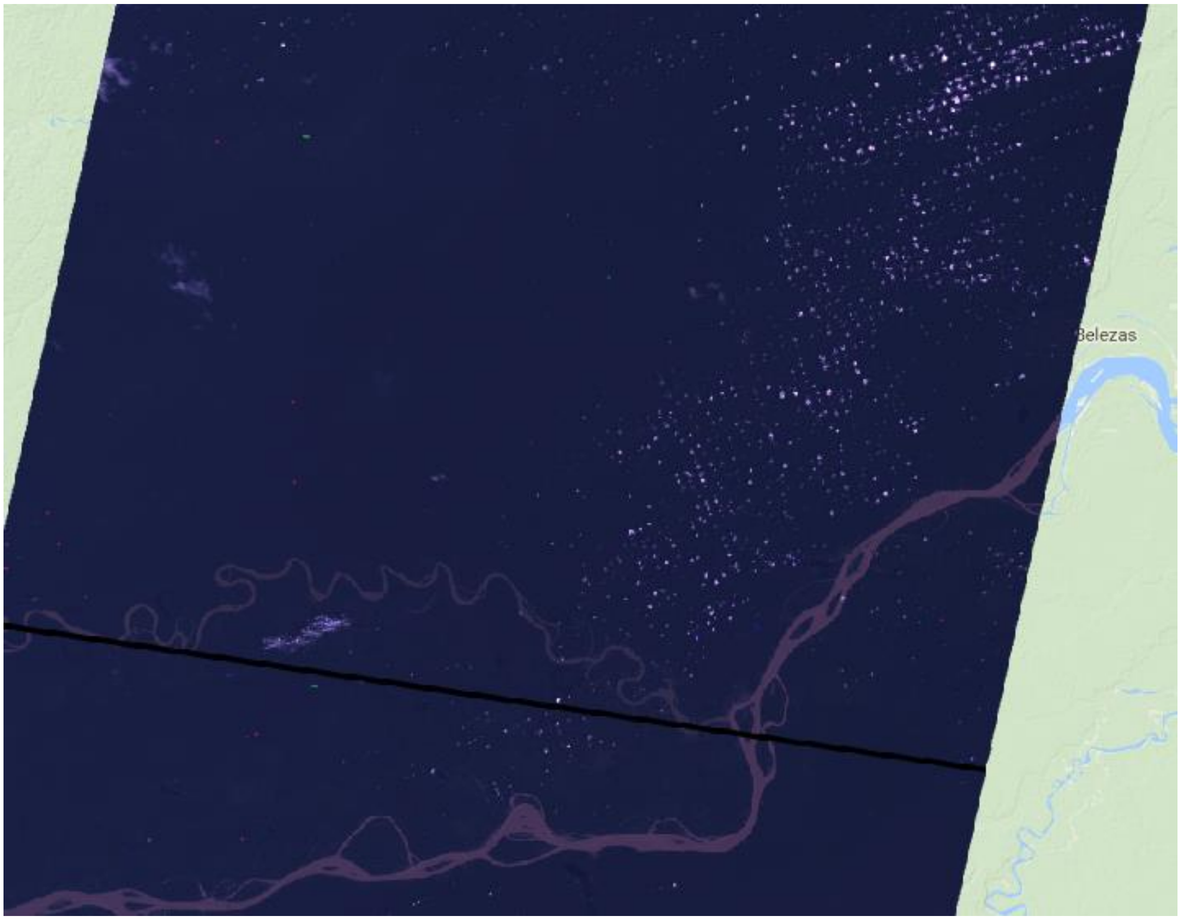

Fig B – A Landsat 5 scene for an 8-day period around Dec 11, 1990. Note the small clouds in the upper right corner of an otherwise clear scene.
